# True Hospitality

**Published:** 1876-02

**Authors:** 


### TRUE HOSPITALITY

Is seldom found, except in country places. We sacrifice reason, and health, and comfort, to pride, pretence, and a vain show. How hateful to our ears are the deceitful apologies made to guests at the dinner-table. In all cases, they are to us a moral emesis, a dose of tartar emetic. How much lack we of that high independence which should characterize the man anywhere—everywhere. We all know that such apologies are falsities in others; but seem to forget that others regard them in the same light as to ourselves, and the meal is commenced with a feeling of disparagement as to our host or hostess, if not of actual contempt; and in proportion to its intensity, does it retard digestion. All feelings at the dinner-table should be of the positively pleasureable kind; in proportion as it is not so, "dining ont" is a bore, and an injury to the body.

To be truly hospitable, make your guest feel himself at home. Let him see that your usual routine is not disturbed; and in in proportion as he is conscious of this, will he feel free to come again, throw off restraint, and make himself at home; feel that he is welcome.

